# Effect of Lectins from *Diocleinae* Subtribe against Oral *Streptococci*

**DOI:** 10.3390/molecules16053530

**Published:** 2011-04-27

**Authors:** Theodora Thays Arruda Cavalcante, Bruno Anderson Matias da Rocha, Victor Alves Carneiro, Francisco Vassiliepe Sousa Arruda, Antônia Sâmia Fernandes do Nascimento, Nairley Cardoso Sá, Kyria Santiago do Nascimento, Benildo Sousa Cavada, Edson Holanda Teixeira

**Affiliations:** 1 Department of Biochemistry and Molecular Biology, Faculty of Medicine of Sobral, Federal University of Ceará, Fortaleza, CE, Brazil; Email: theodorathays@yahoo.com.br (T.T.A.C.); brunoanderson@gmail.com (B.A.M.R.); victorcarneiro@ufc.br (V.A.C.); asamiaf@yahoo.com.br (A.S.F.N.); kyriasantiago@gmail.com (K.S.N); bscavada@gmail.com (B.S.C.); 2 Northeast Biotechnology Network (RENORBIO), State University of Ceará, 60740-000, Fortaleza, CE, Brazil; Email: vassiliepe@gmail.com; 3 LIBS, Integrate biomolecules Laboratory, Faculty of Medicine of Sobral, Federal University of Ceará, Fortaleza, CE, Brazil; Email: nairleysafirmino@gmail.com

**Keywords:** plantlectins, oral *Streptococcus*, biofilm

## Abstract

Surface colonization is an essential step in biofilm development. The ability of oral pathogens to adhere to tooth surfaces is directly linked with the presence of specific molecules at the bacterial surface that can interact with enamel acquired pellicle ligands. In light of this, the aim of this study was to verify inhibitory and antibiofilm action of lectins from the *Diocleinae* subtribe against *Streptococcus mutans* and *Streptococcus oralis*. The inhibitory action against planctonic cells was assessed using lectins from *Canavaliaensi formis* (ConA), *Canavalia brasiliensis* (ConBr), *Canavalia maritima* (ConM), *Canavalia gladiata* (CGL) and *Canavalia boliviana* (ConBol). ConBol, ConBr and ConM showed inhibitory activity on *S. mutans* growth. All lectins, except ConA, stimulated significantly the growth of *S. oralis*. To evaluate the effect on biofilm formation, clarified saliva was added to 96-well, flat-bottomed polystyrene plates, followed by the addition of solutions containing 100 or 200 µg/mL of the selected lectins. ConBol, ConM and ConA inhibited the *S. mutans* biofilms. No effects were found on *S. oralis* biofilms. Structure/function analysis were carried out using bioinformatics tools. The aperture and deepness of the CRD (Carbohydrate Recognition Domain) permit us to distinguish the two groups of *Canavalia* lectins in accordance to their actions against *S. mutans* and *S. oralis*. The results found provide a basis for encouraging the use of plant lectins as biotechnological tools in ecological control and prevention of caries disease.

## 1. Introduction

Many bacteria in Nature often adopt a sessile biofilm lifestyle attached on surfaces and forming matrix-embedded communities called biofilms that differs greatly from that of other free-living cells [1,2]. Such biological organization provides a sheltered micro environment for the immobilized bacteria [3,4]. Those communities have been implicated in many chronic diseases, such as chronic otitis and tonsillitis, in addition to dental caries and periodontal diseases [5]. Microorganisms form a pathogenic biofilm adhered to dental surfaces, producing acid and cytotoxic products that lead to the demineralization of dental tissues [6,7]. Dental caries is a multifatorial disease, both infectious and transmissible, while demineralizing the dental structure [8]. Factors related to diet, host, microbiota, and time of exposition contributes to caries installation and development. Although the application of good oral hygiene practices and fluoridation are generally considered to be responsible for the continuing decline dental caries in industrialized countries, a significant proportion of the population still suffers from tooth decay [9]. 

More than 700 bacterial species have been detected in the oral cavity [10], and the presence of pathogenic bacteria in oral biofilms is a major factor associated with dental diseases such as caries and periodontitis [11,12,13]. For biofilm formation, interactions between bacterial cells and the dental surface have importance for the colonization of the surface at which these communities develop [14]. The first step in this development is the establishment of an acquired enamel pellicle that consists of saliva components, such as proteins, glycoproteins and carbohydrates [15]. The bacteria can interact with the acquired pellicle molecules through a series of specific mechanisms such as lectin interactions involving adhesion between the bacterial surface and the pellicle receptors [16].

Lectins are proteins of non-immune origin that recognize and bind to specific carbohydrate structural epitopes without modifying them [17]. Because of their carbohydrate recognition ability, lectins are involved in several cellular events, beyond symbiotic and pathogenic interaction between microorganisms and hosts [18,19,20]. Leguminous lectins constitute a wide family of homologous proteins, structurally similar and with distinct carbohydrate specificities. They are the most studied and characterized group of plant lectins [21]. However, leguminous lectins have an essentially similar three-dimensional structure, and high amino acid sequence correspondence, yet they show considerable monomer oligomerarization diversity [22,23]. Alterations in the relative orientation of the carbohydrate binding sites at the quaternary structure of homologous lectins have been hypothesized as contributing to the differences in biological activity and potency of *Canavaliaensi formis* and *Canavalia brasiliensis* lectins [24,25].

According with Teixeira and collegues [26] plant lectins are able to inhibit oral bacteria adherence to enamel acquired pellicles probably through blocking streptococci adhesion. This manner, the aim of this study was to verify the activity of legume lectins from Diocleinae subtribe on *Streptococcus mutans* UA 159 and *Streptococcus oralis* ATCC 10557 during planktonic growth and experimental biofilm formation. The results suggest that lectins have anti-adhesion potential and that they can be explored as a biotechnological tool in studies and also in therapeutics of dental diseases that are closely related to biofilm formation.

## 2. Results and Discussion

**Figure 1:**
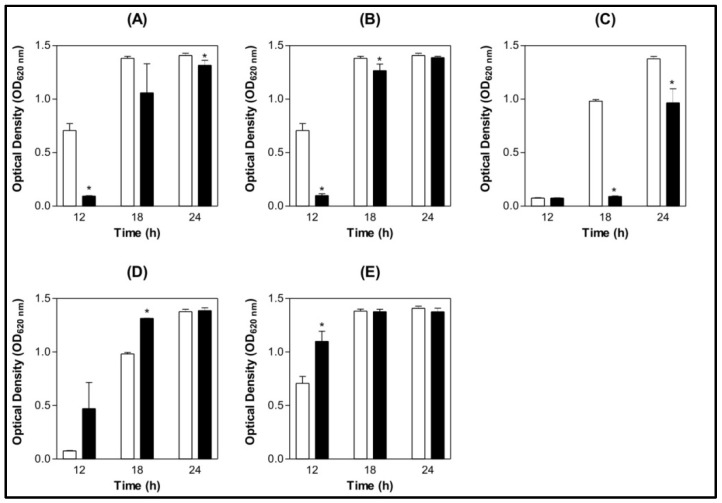
Bar graph of *S. mutans* at different growth time points under the effect of lectins. (**A**) ConBol; (**B**) ConBr; (**C**) ConM; (**D**) CGL; (**E**) ConA.*p < 0.01 related to the 0.9% NaCl.(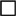) 0.9% NaCl (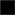) Lectin 500 µg/mL.

Bacterial growth measurements were made at lectin concentrations ranging from 15.6 to 500 µg/mL and for the control (0.9% NaCl). The concentration of 500 µg/mL was chosen due to statistical differences from the control. The other concentrations that were assayed, however, did not demonstrate statistically significant differences compared with the control (data not shown). For *S. mutans*, ConBol, ConBr and ConM showed inhibitory activity compared with the control (Figure 1 A, B and C) and CGL and ConA interestingly stimulated the growth of this bacteria compared to the control (Figure 1 D and E).

**Figure 2:**
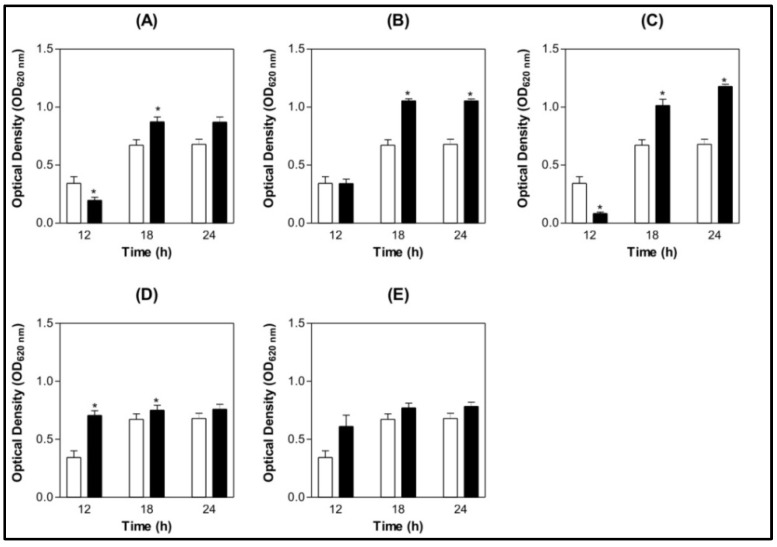
Bar graph of *S. oralis* at different growth time points under the effect of lectins. (**A**) ConBol; (**B**) ConBr; (**C**) ConM; (**D**) CGL; (**E**) ConA.*p <0.01 related to the 0.9% NaCl. (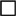) 0.9% NaCl (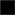) Lectin 500 µg/mL.

Tests of the same lectins against *S. oralis* showed that ConBol, ConBr and CGL (Figure 2 A, B and D) stimulated bacterial growth compared to the control. ConM showed remarkable growth stimulation (Figure 2 C). When ConA was evaluated no action was seen (Figure 2 E).

**Figure 3:**
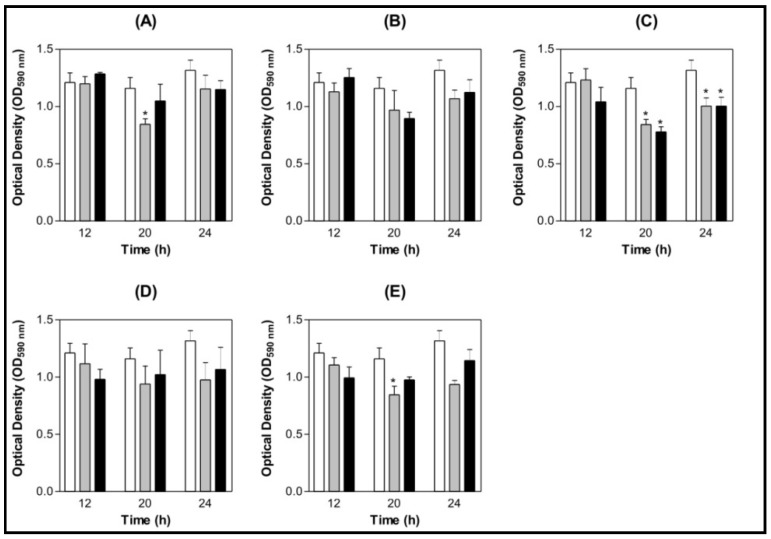
Bar graph of *S. mutans* biofilm content at different growth times under the effect of lectins. The control was Bovine Serum Albumin (BSA) 200 µg/mL. (**A**) ConBol; (**B**) ConBr; (**C**) ConM; (**D**) CGL; (**E**) ConA.*p < 0.01 related to the BSA 200 µg/mL. Legend: (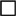) BSA 200 µg/mL (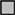) Lectin 100 µg/mL (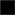) Lectin 200 µg/mL.

**Figure 4:**
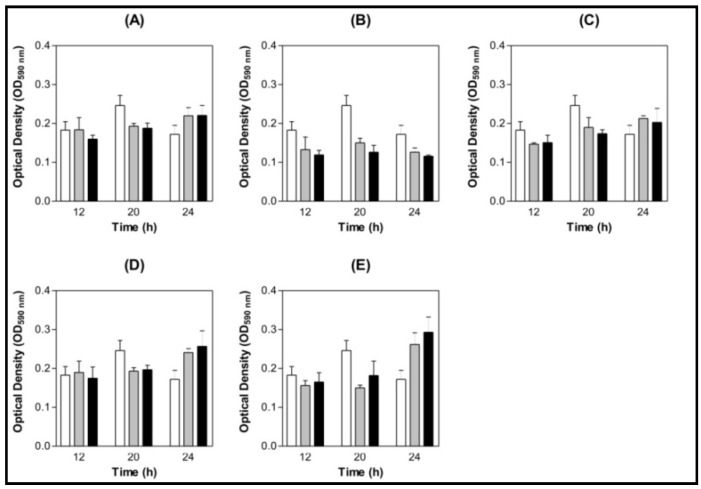
Bar graph of *S. oralis* biofilm content at different growth times under the effect of lectins.The control was Bovine Serum Albumin (BSA) 200 µg/mL. (**A**) ConBol; (**B**) ConBr; (**C**) ConM; (**D**) CGL; (**E**) ConA.*p < 0.01 related to the BSA 200 µg/mL Legend: (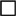) BSA 200 µg/mL (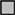) Lectin 100 µg/mL (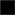) Lectin 200 µg/mL.

ConBol and ConA prevented *S. mutans* biofilm formation when the concentration tested was 100 µg/mL and ConM showed the same effect even at 100 µg/mL as 200 µg/mL (Figure 3 A, C and E). No effects were found when ConBr and CGL were tested (Figure 3 B and D). No inhibition or stimulation effects were found on *S. oralis* biofilms (Figure 4 A to E). All statistical analyses were made comparing to a BSA control.

**Figure 5:**
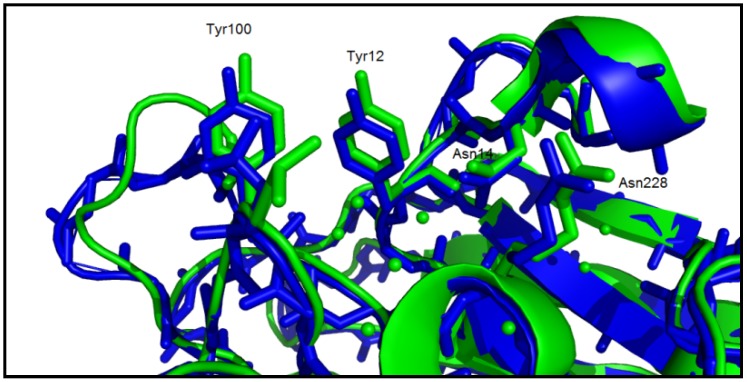
Comparison between CGL (stimulatory lectin for *S. mutans* - blue) and ConM (inhibitory lectin for *S. mutans* - green) carbohydrate binding sites. The distances between the amino acids side chains can explain the divergent activity caused by the interaction pattern with surface carbohydrates.

The bacteria growth inhibition assays revealed that *S. mutans* was inhibited by ConBol, ConBr, and ConM, while ConA and CGL stimulated the growth of *S. mutans* when compared with the control. The carbohydrate binding site of these lectins is quite similar and diverges slightly from ConA and CGL; this divergence is mainly linked to distances between amino acid side chains which compose the carbohydrate recognition domain. The main difference observed in the CRD (Carbohydrate Recognition Domain) was a reduction of the deepness of the site (Arg228-Tyr12 and Arg228-Asn14) and of the aperture (Tyr100–Tyr12 and Tyr12–Tyr14) (Figure 5). The aperture and deepness of the CRD permit to distinguish two groups of *Canavalia* lectins in accordance to their actions against *S. mutans*; the inhibitory group formed by ConBol, ConBr and ConM and the stimulatory group composed of ConA and CGL.

The first report of inhibitory action of peptides against microorganisms dates from 1942, and refers to a protein obtained from wheat flour [27]. Lectins in higher plants defend against pathogenic bacteria and fungi by recognizing and immobilizing the infecting microorganisms via binding, thereby preventing their subsequent growth and multiplication [28]. The inhibition of bacteria and fungi by lectins, such as those of the herbaceous *Amaranthus*, has long been known [29].

The present work aimed to assess antibacterial action and inhibition of biofilm formation by leguminous lectins extracted from the *Diocleinae* subtribe. Determination of antimicrobial action following NCCLS guidelines suggested that in the concentrations used and under the conditions stated, only ConBol, ConBr and ConM had interference at 500 µg/mL diminishing *S. mutans* growth. CGL and ConA had a stimulatory effect against this bacterium. However, at the tests of *S. oralis* all lectins, except ConA, had a stimulatory effect at the same concentration, 500 µg/mL.

The concentration used is quite high compared to the concentrations used in similar studies [30,31,32,33]. However, Liao and collegues [30], testing antimicrobial action of plant and marine algae lectins used concentrations ranging from 102 to 800 µg/mL and found that Con A and WGA from land plants did not inhibit any of the vibrios assayed. 

Our results showed that ConM was able to interfere in *S. mutans* growth, what can be attributed to cellular membrane damage. Santi-Gadelha and collegues [31] showed though electron microscopy the presence of pores and severe disruption of the Gram-positive bacteria membrane demonstrating strong antimicrobial activity and pointing a possible mechanism of inhibition of bacterial growth by lectins. This pores formed at the membrane permit outflowing of cellular contents [33,34]. 

The carbohydrate-binding site at the bacterial surface, probably plays a key role in this antibacterial activity, being responsible for the recognition of bacteria. It is important to note that differences in antimicrobial action between *S. mutans* and *S. oralis* may be due to differences in characteristic carbohydrate surface composition. Almost all microorganisms express surface-exposed carbohydrates. These carbohydrates may be covalently bound, as in glycosylated teichoic acids to peptidoglycan, or non-covalently bound, as in capsular polysaccharides [31,35]. Every surface-exposed carbohydrate is a potential lectin-reactive site. The ability of lectins to form complexes with microbial glycoconjugates has made it to be employed as probes and sorbents for whole cells, mutants, and numerous cellular constituents and metabolites. Microbial receptors for concanavalin A have been described. For example, polyelectrolyte glucosylated teichoic acid found in many Gram-positive bacteria surfaces [35] and neutral polysaccharides produced by members of the genera *Leuconostoc* and *Streptococcus* [31]. The development of high-affinity ligands capable of selectively recognizing a variety of small motifs in different oligosaccharides would be of significant interest as experimental and diagnostic tools for several bacterial infections. The selective binding of certain lectins to bacteria has been proposed for targeted delivery of antimicrobial agents with *C. ensiformis* lectin targeting *Streptococcus sanguis* and *Corynebacterium hofmannii* and *Triticum vulgaris* lectin targeting *Streptococcus epidermis* “*in vitro*” [36]. This could be a new perspective area for future studies involving the conjugation of lectins and other products with known bactericidal action.

The resident microflora benefits the host by acting as part of the host defences and preventing colonization by exogenous (and often pathogenic) microorganisms, “colonization resistance” [37]. The concept of microbial ecological change as a mechanism to prevent dental change is an important one [38]. Our results showed that the lectins of *C. boliviana*, *C. brasiliensis* and *C. maritime* had interference effects against *S. mutans*, and a stimulatory effect on *S. oralis* growth. 

*S. mutans*, an acidogenic bacterium, has been associated with the development of tooth decay, through acid production as a result of carbohydrate fermentation [39]. Microorganisms present at the oral cavity like *S. oralis* are numerically important members of the oral microbiota, isolated from all intraoral surfaces and are pioneering organisms involved in primary colonization at dentition [40,41]. Marsh [42], in the “ecological plaque hypothesis”, proposed that changes in environmental factors trigger a shift in the balance of the resident microbiota, and this could predispose a site to disease.

One of many molecular mechanisms involved in adherence of bacteria and the development of mixed-species oral biofilms is specific lectin-carbohydrate interactins between bacteria, such as the interaction involved in lactose-sensitive congregations of *Actinomyces* spp. and *Streptococcus* spp. Because of their role in adhesion and agglutination, lectins are considered to be important in both symbiotic and pathogenic interactions between microorganisms and hosts [32]. With the new understanding of oral microbial community interactions, there is now interest in approaches that selectively inhibit oral pathogens, or modulate the microbial composition of dental plaque to control community based microbial pathogenesis [43].

Our results indicate an inhibition on *S. mutans* biofilm formation and not the same effect to *S. oralis*. According to Islam and colleagues, this could be attributed to the surface carbohydrate differences between the bacteria species. Lectins agglutinate bacteria and their presence in the milieu can be exploited to prevent the long-term attachment of the bacteria on to the tooth surface [44]. Liljemark and collaborators [45] showed the effect of bacterial aggregation on the adherence of oral streptococci to acquired pellicle using lectins. Their results suggest that the formation of large aggregates causes a decrease of the number of adherent bacteria and indicates that independent of a certain lectin concentration, its aggregating activity does not oppose to its blocking effect and even helps decrease the number of adherent streptococci. Significant inhibition of bacterial biofilm growth caused by plant lectins was reported before by Islam and colleagues [44], a fact that can be linked to a surface glycoprotein of *S. mutans* of 60 kDa (with mannose and *N*-acetylgalactosamine) that has been known to be involved in saliva and bacterial interaction [46]. The lesser adherence in the presence of glucose/mannose specific lectins could be because of the interaction with this protein [44].

The differences in the pathway of lectin antimicrobial action are related to quaternary assembly but recent crystallographic studies reveal that the specific carbohydrate binding site orientation determines the biological response of many systems to the presence of the lectin [47,48]. The stimulatory effect of *Canavalia* lectins on *S. oralis* might be due the high frequency of neuraminic acid at cell surface [49], which is not specific to binding *Diocleinae* lectins.

The inhibition of *S. mutans* caused by three lectins out of the five tested is structurally confirmed by the comparison of amino acid side chain distances at the carbohydrate recognition domain (mainly the aperture versus the deepness of the site). These aspects also have been reported for ConM and CGL to increase the selectivity of lectins for disaccharides [47,48,50]. The preference of ConBol, ConBr and ConM to bind and inhibit specifically *S. mutans* strain can be explained by the reduction of the primary carbohydrate binding site and the increase of the specificity of these lectins by specific carbohydrate epitopes, which form more complex interaction patterns with the tested lectins despite ConA and CGL presenting larger primary carbohydrate binding sites. Quantitative differences ant interaction path can exist between lectins that recognize the same terminal monosaccharide residues [51]. Beside this, the same lectins can be more specific for complex carbohydrate structures than for monosaccharide residues [52]. Since the mechanisms of action of leguminous lectins on bacteria cells still remains unknown, this study provides new insights on the molecular basis of lectin-bacteria interactions. 

## 3. Experimental

### 3.1. Bacterial Strains and Growth Conditions

Bacterial species *Streptococcus mutans* UA 159 and *Streptococcus oralis* ATCC 10557 used in this study were obtained from the Instituto Oswaldo Cruz–FIOCRUZ collection. The strains were kept in BHI (Brain Heart Infusion–Difco) and glycerol 20% at −80 °C; for experimental procedures, 100 µL were inoculated in 10 mL of fresh BHI broth and incubated for 18 h, with CO_2_ 10% at 37 °C. After initial activation, the culture was renewed in 10 mL of sterile BHI broth adding 100 µL of inoculum and grown under the same conditions described above.

### 3.2. Lectin Purification

The lectins from *Canavalia ensiformis* (ConA) [53], *Canavalia brasiliensis* (ConBr) [54], *Canavalia maritima* (ConM) [55], *Canavalia gladiata* (CGL) [56] and *Canvalia boliviana* (ConBol) [57], were obtained at Biologically Active Molecules Laboratory (Biomol - LAB), Department of Biochemistry and Molecular Biology, Science Center, Federal University of Ceará-UFC, according to its respective purification references.

Briefly, mature seeds were collected at Ceará State, Northeast Brazil. The seeds were ground to a fine powder in a coffee mill and then defatted with *n*-hexane. Soluble proteins were extracted at 20 °C for 4 h by continuous stirring with 0.9% NaCl (1:10 w/v), followed by centrifugation at 10,000 g at 4 °C for 20 min. The supernatant was applied to a Sephadex G-50 column (5 × 25 cm), which had been equilibrated with 0.9% NaCl containing 5 mmol/L CaCl_2 _and MnCl_2_. The column was then washed with equilibration buffer at a flow rate of 45 mL/h until the effluent absorbance at 280 nm was below 0.05. The bound lectin was eluted with 0.1 mol/L glycine, pH 2.6, dialyzed extensively against distilled water and lyophilized. The affinity chromatography fraction was further purified using an Äkta chromatographic system and Mono-Q column (5 × 0.5 cm) equilibrated with 20 mmol/L Tris-HCl pH 7.0, and developed with a linear gradient of 20 mmol/L Tris-HCl pH 7.0, containing 1.0 mol/L NaCl at a flow rate of 1 mL min-1 and a slope of 5% NaCl/min. The lectin preparations, recovered in the unbound fraction, were exhaustively dialyzed against distilled water and freeze-dried. The purity of lectins was assessed by SDS-PAGE.

### 3.3. Effect of Lectins on Bacterial Growth

After cell growth, the culture was centrifuged at 1.500 g for 20 min at 4 °C, washed twice with 0.9% NaCl and then adjusted for a concentration of 10^8^–10^9^ Colony Forming Units (CFU)/mL. The lectin solutions were prepared by 2-fold serial dilution (concentrations ranging from 15.6 to 500 µg/mL) and incubated together with the selected adjusted bacteria for 6 h with 10% CO_2_ at 37 °C, without culture broth to avoid possible binding of the lectins to available carbohydrate at the medium. This procedure was based on growth curve experiments with these bacteria on 0.9% NaCl observing the time point of cell viability decrease through bacterial plating each 30 min (Data not show). The assay in polystyrene plates was carried out by adding 200 µL of fresh BHI broth to each well and 4 µL of the lectin/bacteria suspension previously incubated at each concentration. The samples were made in triplicate with three separated experiments. The control used was 0.9% NaCl, which represents the normal growth of bacteria using the same procedure for bacteria/lectin incubation. When the plates were ready, an initial measurement at OD_620nm_ (Biotrak II Plate Reader–Amersham Biosciences) was carried out to establish a baseline. Other measurements were made after 12, 18 and 24 h of incubation at 37 °C with 10% CO_2_.

### 3.4. Effect of Lectins on S. mutans and S. oralis Biofilm Formation on Saliva-coated Surface

#### 3.4.1. Saliva Processing

Saliva was collected and processed according to the protocol of Guggenheim and colleagues [58]. Briefly, whole unstimulated saliva was collected for 1 h per day during several days from volunteers that assigned an informed consent term (Ethical Committee Approval number 217-CONEP/CNS/MS) at least 1.5 h after eating, drinking, or tooth cleaning. Saliva samples were collected in sterile 50 mL polypropylene tubes, chilled in an ice bath or frozen at −20 °C. After 500 mL saliva had been collected, it was pooled and centrifuged (30 min, 4 °C, 27.000 g); the supernatant was pasteurized (60 °C, 30 min) and re-centrifuged in sterile tubes. The resulting supernatant was stored into sterile 50 mL polypropylene tubes at −80 °C. The efficiency of the process was assessed by plating processed saliva samples onto BHI agar; after 72 h at 37 °C no CFUs were observed on incubated plates.

#### 3.4.2. Biofilm Assay

For plate mounting 200 µL of processed saliva was added to each well for 2 h under agitation (MA-420-Marconi orbital shaking incubator board) at 37 °C. Then, the saliva was removed and 200 µL of lectins solution at 100 or 200 µg/mL and bovine serum albumin (BSA) control (200 µg/mL) were added for 2 h at 37 °C. After this period, the solutions were removed from the wells and 200 µL of a bacterial suspension adjusted to a concentration of 10^6^–10^8^ CFU/mL were placed remaining for 2 h. In sequence, the bacterial solution was removed and 200 µL of sterile BHI broth was added and the plate incubated at 37 °C, 10% CO_2_. Every step of the experimental protocol was followed by washing procedure with 200 µL of 0.9% NaCl, with up and down movements. As control it was used BSA [31] to observe the action of a protein without antimicrobial action on the medium.

After incubation for 12, 20 and 24 h at 37 °C with 10% CO_2_, the media was removed from the microtitre plates. The remaining planktonic cells were removed by gentle washing with sterile water. The wells with the adhered biofilms were fixed with formalin (37%, diluted 1:10) plus 2% sodium acetate for 15 min, and stained with 200 µL of 0.1% crystal violet for 15 min at room temperature. After two rinses with distilled water, remaining (bound) dye was removed with 100 µL of 95% ethanol. The plates were then placed in agitation for 5 min to allow full release of the dye. The biofilm formation was then quantified by measuring the optical density at 570 nm by ELISA plate reader (Biotrak II Plate Reader–Amersham Biosciences) [59]. A total of tree plates were mounted for each bacterium and different concentration (12, 20 ad 24 h of incubation) by experiment. The overall experiment was repeated three times.

### 3.5. Structure/Function Analysis

Atomic coordinates for the lectins crystal structures from the Protein Data Bank (PDB) were used to compare the carbohydrate binding sites design, and relate them to biological activity. The PDB codes for the proteins used in the analysis were: 1WUV for *Canavalia gladiata* [47], 2CWM for *Canavalia maritime* [50], *Canavaliaensi formis* (PDB code 1JBC) [53], *Canavalia brasiliensis* (PDB code 3JU9) [54] and *Canavalia boliviana* [57]. The structural analyses were performed using PyMol [60].

### 3.6. Statistical Analysis

The data are presented as means ± S.E.M and compared using ANOVA and Bonferroni post hoc test. *p* < 0.01 was considered to be statistically significant.

## 4. Conclusions

Evaluating all plant lectins tested it was observed that ConM was effective in improving *S. oralis* growth and diminishing *S. mutans* biofilm development. This mode of action can led to a new mode of dental caries control by biological manipulation of dental sites. Substances that can minimize these communities’ way of life can emerge as a new possibility for biotechnological therapy for patients with high streptococci numbers by acting as a biological treatment. Our results indicate a new pathway for prevention of caries disease.
